# Long-Term Risk of Being Bedridden in Elderly Patients Who Underwent Oncologic Surgery: A Retrospective Study Using a Japanese Claims Database

**DOI:** 10.1245/s10434-023-13566-5

**Published:** 2023-05-06

**Authors:** Takaaki Konishi, Yusuke Sasabuchi, Hiroki Matsui, Masahiko Tanabe, Yasuyuki Seto, Hideo Yasunaga

**Affiliations:** 1 Department of Breast and Endocrine Surgery, Graduate School of Medicine, The University of Tokyo, Tokyo, Japan; 2 Department of Clinical Epidemiology and Health Economics, School of Public Health, The University of Tokyo, Tokyo, Japan; 3 Data Science Center, Jichi Medical University, Shimotsuke, Tochigi Japan

## Abstract

**Background:**

Although functional outcomes are important in surgery for elderly patients, the long-term functional prognosis following oncologic surgery is unclear. We retrospectively investigated the long-term, functional and survival prognosis following major oncologic surgery according to age among elderly patients.

**Methods:**

We used a Japanese administrative database to identify 11,896 patients aged ≥ 65 years who underwent major oncological surgery between June 2014 and February 2019. We investigated the association between age at surgery and the postoperative incidence of bedridden status and mortality. Using the Fine–Gray model and restricted cubic spline functions, we conducted a multivariable, survival analysis with adjustments for patient background characteristics and treatment courses to estimate hazard ratios for the outcomes.

**Results:**

During a median follow-up of 588 (interquartile range, 267–997) days, 657 patients (5.5%) became bedridden and 1540 (13%) died. Patients aged ≥ 70 years had a significantly higher incidence of being bedridden than those aged 65–69 years; the subdistribution hazard ratios of the age groups of 70–74, 75–79, 80–84, and ≥ 85 years were 3.20 (95% confidence interval [CI], 1.53–6.71), 3.86 (95% CI 1.89–7.89), 6.26 (95% CI 3.06–12.8), and 8.60 (95% CI 4.19–17.7), respectively. Restricted cubic spline analysis demonstrated an increase in the incidence of bedridden status in patients aged ≥ 65 years, whereas mortality increased in patients aged ≥ 75 years.

**Conclusions:**

This large-scale, observational study revealed that older age at oncological surgery was associated with poorer functional outcomes and higher mortality among patients aged ≥ 65 years.

**Supplementary Information:**

The online version contains supplementary material available at 10.1245/s10434-023-13566-5.

The prevalence of malignant tumors is increasing, especially in elderly patients aged ≥ 65 years; two-thirds of malignant solid tumors are diagnosed in elderly patients.^1,2^ When considering indications for surgery, which is a standard treatment in this population, frail and numerous comorbidities can be a major concern.^2–5^ Additionally, general life expectancy and survival prognosis of malignancy should be considered in the decision-making process of treatments.^1,5^ Clinicians do not necessarily perform oncologic surgery due to underestimation of the general life expectancy in elderly patients, although the life expectancy has been extended.^2,5^ Specifically, Japan had the longest life expectancy at the age of 65 years (20.1 years for men and 24.9 years for women in 2020).^6^

Impaired activity of daily living following surgery also is a serious concern among the elderly.^7,8^ The impaired activity would result in poor quality of life; moreover, if a patient becomes bedridden, the condition is accompanied by several complications, such as bedsores, deep venous thrombosis, pneumonia, and urinary tract infections, deteriorating the optimal allocation of medical resources.^9–12^ Because poor functional capacity will have a negative impact on survival, maintenance of function and independence has become one of the major principles of cancer management in the elderly.^13^ Furthermore, many elderly patients prioritize functional prognosis over survival prognosis; that is, they do not prefer undergoing surgery to prolong overall survival if the surgery worsens the activities of daily living.^14^ Thus, assessment of the risk of impaired postoperative activity of daily living is important in the treatment selection for the elderly. However, although one study reported in-hospital postoperative activity in the elderly, long-term postoperative activity remains unclear.^8^

Hence, we investigated the long-term functional and survival prognosis after major oncologic surgery according to age in elderly patients using a large-scale administrative database in Japan, one of the most aged countries.^15^

## Methods

### Data Source

We conducted this retrospective cohort study by using the data from an administrative database of 18 of 25 districts in Tochigi Prefecture, a northern prefecture of the Greater Tokyo Area.^16^ The database contains claims of two medical insurances and a long-term care insurance of approximately 1,300,000 residents, representing approximately 60% of residents in the prefecture. The two types of medical insurances were National Health Insurance (for self-employed individuals, retired individuals, and their dependents) and Late Elders’ Health Insurance (for all people aged ≥ 75 years of age). Because almost everyone in Japan has been publicly insured for medical care since 1961,^17,18^ almost all residents aged ≥ 65 years in the 18 districts were included in the database. In addition, the Japanese government implemented mandatory public long-term care insurance in 2000.^19–21^ Residents aged ≥ 65 years are regarded as insured candidates, and candidates who meet the eligibility criteria can receive long-term care insurance based on their care-need level. Accordingly, this database contains the following patient-level data for hospitalization and outpatient visits: date of birth, sex, diagnoses recorded using the International Classification of Diseases, 10th revision (ICD-10) codes, interventions and surgical procedures, care-need level, and death.

The care-need level is assessed by a nationally standardized certification system, irrespective of the income level and availability of informal care provided by the family.^19^ First, a trained, local, government official visits a candidate (at home or hospital) to evaluate nursing care needs using a questionnaire of approximately 90 items on the current physical and mental status and daily use of medical services with a detailed note. Concurrently, the attending primary care physician fills out a paper-based statement on the candidate’s condition in a common format. Subsequently, the results of the questionnaire and part of the statement are computed to assign the candidate to one of the seven care-need levels based on the total daily estimated care minutes: support level 1, 25–31 min; support level 2, 32–49 min; care-need level 1, 32–49 min; care-need level 2, 50–69 min; care-need level 3, 70–89 min; care-need level 4, 90–109 min; and care-need level 5, ≥ 110 min. Finally, the Nursing Care Needs Certification Board, which includes physicians, nurses, and other experts in health and social services appointed by a mayor, determines the final care-need level that determines service benefits covered by long-term care insurance after considering the note by the officer and the statement by the primary care physician. In principle, the care-need level is reevaluated once or twice per year. A previous study reported that the level correlates highly with the Barthel index,^22^ support levels 1–2 and care-need level 1 were comparable with Barthel index scores of 85–95 (independent with minor assistance), care-need levels 2–3 were comparable with Barthel index scores of 65–80 (partial dependence), and care-need levels 4–5 were comparable with Barthel index scores of < 40 (complete dependence, bedridden).

### Study Protocol

Using the aforementioned administrative database, we retrospectively identified patients aged ≥ 65 years who underwent major oncologic surgery between June 2014 and February 2019. We considered oncologic surgeries for the bladder, breast, colorectum, stomach, liver, lung, pancreas, prostate, and uterus as major oncologic surgeries. We used the original Japanese procedure codes for surgery to identify the patients. We excluded patients who (1) were bedridden (care-need levels 4–5) before surgery (because they were unable to experience the primary outcome) and (2) were included in the database within 6 months preceding surgery (window period). We categorized the eligible patients into five groups according to their age at surgery: 65–69, 70–74, 75–79, 80–84, and ≥ 85 years.

The primary outcome was the incidence of being bedridden (care-need levels 4–5). The secondary outcome was mortality.

We examined background factors, including patient characteristics (sex, comorbidities, and care-need level before surgery) and treatment background (site of surgery, preoperative treatments, surgery on the day of admission, scopic surgery, and in-hospital rehabilitation). Comorbidities were assessed by using the Charlson comorbidity index, which was defined by the diagnosis of ICD-10 codes within the window period (i.e., 6 months preceding surgery).^23,24^ Regarding preoperative treatments, we investigated whether chemotherapy and radiotherapy were conducted within the window period.

The requirement for informed consent in the present study was waived because of the anonymity of the patient database. This study followed the Strengthening the Reporting of Observational studies in Epidemiology statement and was approved by the Institutional Review Board of The Jichi Medical University (approval number 17-002; September 4, 2017).^25^

### Statistical Analysis

First, we described the number of outcomes and depicted the Kaplan–Meier curve for outcomes stratified by age category. The follow-up started at surgery and ended at the incidence of the outcomes, exit from the database, or February 2019, whichever occurred first. Additionally, we depicted the Kaplan–Meier curve for mortality in patients who were bedridden postoperatively. The follow-up started when the patient became bedridden and ended at death, exit from the database, or February 2019, whichever occurred first.

Second, we conducted multivariable survival analyses to investigate the association between age category and outcomes, with adjustment for the aforementioned background factors (patient characteristics and treatment background). Hazard ratios were obtained as estimates of the relative risk of outcomes with reference to patients aged 65–69 years, using Cox proportional hazards regression models. In the analysis for bedridden status, we used a Fine–Gray subdistribution hazard model.^26,27^ A conventional survival analysis model is based on the assumption that a patient can be censored by an event independent of the outcome of interest (noninformative censoring). However, in the current study, if a patient dies, the patient will not become bedridden; that is, censoring by mortality prevents the occurrence of being bedridden (informative censoring by competing risk). Thus, we used the Fine–Gray model, in which mortality was considered as a competing risk.

Third, we used restricted cubic spline functions to investigate nonlinear associations between age and outcomes. This function enables continuous variables (such as age) to be analyzed without categorization, whereas general regression analysis requires the categorization of continuous variables, which can result in the loss of information and statistical power.^28,29^ In this study, we used four points (70, 75, 80, and 85 years) as the knots in the cubic splines to allow for nonlinear continuous dose–response effects of age. The splines were restricted to linear below the first knot and above the last knot. Hazard ratios with reference to the age of 65 years were calculated using multivariable survival analysis with restricted cubic spline functions. The spline curve was constructed using the “xbrcspline” and “mkspline” commands in Stata (StataCorp LLC, College Station, TX).

Finally, we performed sensitivity analyses: analysis stratified by site of surgery and analysis in patients with no preoperative care-need level. In the stratified analysis, we defined surgery for the colorectum, stomach, liver, and pancreas as digestive surgery in accordance with a previous study.^30^ The other surgeries were defined as nondigestive surgery. We conducted restricted cubic spline functions during sensitivity analyses.

In summary statistics, we used chi-squared test for categorical variables. All 95% confidence intervals (CIs) and *p*-values were based on two-sided hypothesis tests; *p* < 0.05 was considered to denote statistical significance. We conducted statistical analyses by using Stata/SE 17.0 (StataCorp, College Station, TX) software.

## Results

We identified 13,963 patients aged ≥ 65 years who underwent major oncological surgery between June 2014 and February 2019. We excluded 2067 patients who (1) were bedridden before surgery (*n* = 143) and (2) were included in the database within the window period (*n* = 1924). Of the eligible 11,896 patients, 2499 patients (21%) were aged 65–69 years, 2965 (25%) aged 70–74 years, 3018 (25%) aged 75–79 years, 2023 (17%) aged 80–84 years, and 1391 (12%) aged ≥ 85 years at surgery.

In total, male sex was dominant (62%; Table 1). Patients aged ≥ 85 years had a lower Charlson comorbidity index and higher care-need level and were more likely to receive conventional surgery and in-hospital rehabilitation compared with younger patients. Regarding the site of surgery, surgery for the bladder, colorectum, and uterus was performed in approximately a quarter of the total patients; these surgeries were more common among older patients. Older patients were less likely to undergo surgery for the breast, liver, lung, and prostate.

**Table 1:** Background characteristics of 11,896 elderly patients who underwent major surgery categorized by age category

	Age category (yr)					Total	*P* value
	65–69	70–74	75–79	80–84	≥ 85		
	*n* = 2499	*n* = 2965	*n* = 3018	*n* = 2023	*n* = 1391	*n* = 11,896	
Patient characteristics							
Male sex	1540 (62)	1912 (64)	1960 (65)	1220 (60)	742 (53)	7374 (62)	< 0.001
CCI							< 0.001
2	207 (8.3)	243 (8.2)	230 (7.6)	209 (10)	173 (12)	1062 (8.9)	
3	208 (8.3)	257 (8.7)	264 (8.7)	203 (10)	178 (13)	1110 (9.3)	
4	161 (6.4)	211 (7.1)	240 (8.0)	180 (8.9)	182 (13)	974 (8.2)	
5	85 (3.4)	133 (4.5)	138 (4.6)	103 (5.1)	95 (6.8)	554 (4.7)	
6	48 (1.9)	75 (2.5)	80 (2.7)	84 (4.2)	57 (4.1)	344 (2.9)	
7	23 (0.9)	38 (1.3)	46 (1.5)	29 (1.4)	23 (1.7)	159 (1.3)	
8	365 (15)	307 (10)	304 (10)	166 (8.2)	109 (7.8)	1251 (11)	
9	502 (20)	544 (18)	512 (17)	289 (14)	156 (11)	2003 (17)	
10	387 (15)	490 (17)	471 (16)	295 (15)	154 (11)	1797 (15)	
11	261 (10)	298 (10)	314 (10)	201 (9.9)	109 (7.8)	1183 (9.9)	
12	125 (5.0)	164 (5.5)	176 (5.8)	112 (5.5)	63 (4.5)	640 (5.4)	
13	54 (2.2)	84 (2.8)	101 (3.3)	80 (4.0)	50 (3.6)	369 (3.1)	
14	31 (1.2)	40 (1.3)	55 (1.8)	32 (1.6)	21 (1.5)	179 (1.5)	
≥ 15	42 (1.7)	81 (2.7)	87 (2.9)	40 (2.0)	21 (1.5)	271 (2.3)	
Care-need level							< 0.001
None	2456 (98)	2870 (97)	2802 (93)	1706 (84)	897 (64)	10,731 (90)	
Support level 1	11 (0.4)	22 (0.7)	50 (1.7)	75 (3.7)	93 (6.7)	251 (2.1)	
Support level 2	7 (0.3)	20 (0.7)	52 (1.7)	64 (3.2)	87 (6.3)	230 (1.9)	
Care-need level 1	9 (0.4)	21 (0.7)	41 (1.4)	98 (4.8)	133 (9.6)	302 (2.5)	
Care-need level 2	7 (0.3)	18 (0.6)	45 (1.5)	47 (2.3)	116 (8.3)	233 (2.0)	
Care-need level 3	9 (0.4)	14 (0.5)	28 (0.9)	33 (1.6)	65 (4.7)	149 (1.3)	
Treatment background							
Site of surgery							
Bladder	394 (16)	583 (20)	674 (22)	560 (28)	516 (37)	2727 (23)	< 0.001
Breast	382 (15)	342 (12)	316 (10)	188 (9.3)	122 (8.8)	1350 (11)	< 0.001
Colorectum	590 (24)	668 (23)	827 (27)	620 (31)	462 (33)	3167 (27)	< 0.001
Stomach	301 (12)	395 (13)	431 (14)	353 (17)	209 (15)	1689 (14)	< 0.001
Liver	60 (2.4)	79 (2.7)	77 (2.6)	33 (1.6)	12 (0.9)	261 (2.2)	0.001
Lung	278 (11)	387 (13)	382 (13)	179 (8.8)	52 (3.7)	1278 (11)	< 0.001
Pancreas	92 (3.7)	139 (4.7)	133 (4.4)	76 (3.8)	14 (1.0)	454 (3.8)	< 0.001
Prostate	343 (14)	353 (12)	165 (5.5)	19 (0.9)	4 (0.3)	884 (7.4)	< 0.001
Uterus	527 (21)	765 (26)	915 (30)	691 (34)	563 (40)	3461 (29)	< 0.001
Preoperative treatments							
Chemotherapy	226 (9.0)	227 (7.7)	197 (6.5)	73 (3.6)	62 (4.5)	785 (6.6)	< 0.001
Radiotherapy	36 (1.4)	45 (1.5)	44 (1.5)	30 (1.5)	13 (0.9)	168 (1.4)	0.62
Same-day admission*	69 (2.8)	79 (2.7)	70 (2.3)	90 (4.4)	69 (5.0)	377 (3.2)	< 0.001
Scopic surgery	825 (33)	976 (33)	939 (31)	512 (25)	245 (18)	3497 (29)	< 0.001
In-hospital rehabilitation	527 (21)	765 (26)	915 (30)	691 (34)	563 (40)	3461 (29)	< 0.001

*CCI* Charlson comorbidity index

*Surgery on the day of admission

**Fig. 1:**
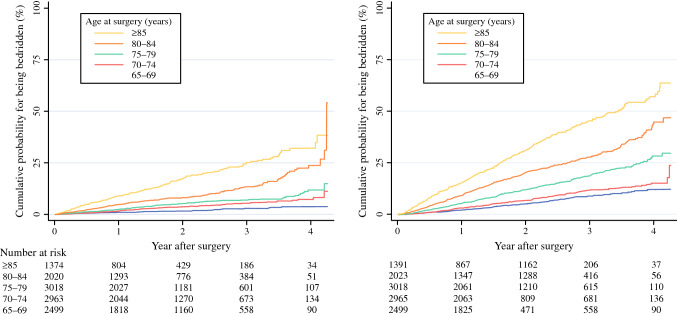
Kaplan–Meier curves for outcomes following oncologic surgery categorized by age at surgery

In total, 657 (5.5%) patients became bedridden, and 1540 (13%) died within a median follow-up period of 588 (interquartile range, 267–997) days (Table 2). The Kaplan–Meier curve for outcomes categorized by age at surgery is shown in Fig. 1. Older patients had consistently higher incidence of adverse outcomes. Patients aged ≥ 85 years had a higher incidence of being bedridden immediately after surgery than younger patients. Additionally, patients aged 80–84 years demonstrated an increasing probability of being bedridden, especially 3 years after surgery. Patients aged ≥ 75 years showed a higher cumulative probability of mortality than those aged 65–69 years and 70–74 years. In patients aged ≥ 80 years, the cumulative probability of mortality was approximately 50%. Of the patients who were bedridden postoperatively, 436 (66%) died within a median period of 62 (interquartile range, 11–184) days after becoming bedridden (Supplementary Table 1). The Kaplan–Meier curve demonstrated that patients aged 65–69 years at surgery died earlier than those aged ≥ 70 years (Supplementary Fig. 1).

**Table 2:** Outcomes of 11,896 elderly patients who underwent major surgery categorized by age category

	Age category (yr)					Total	*P* value
	65–69	70–74	75–79	80–84	≥ 85		
	*n* = 2499	*n* = 2965	*n* = 3018	*n* = 2023	*n* = 1391	*n*=11,896	
Bedridden	45 (1.8)	102 (3.4)	139 (4.6)	174 (8.6)	197 (14)	657 (5.5)	< 0.001
Mortality	137 (5.5)	207 (7.0)	363 (12)	402 (20)	431 (31)	1540 (13)	< 0.001

These outcomes occurred within a median follow-up period of 588 (interquartile range, 267–997) days. The Kaplan–Meier curves for the cumulative probabilities of these outcomes are shown in Fig. 1

**Fig. 2:**
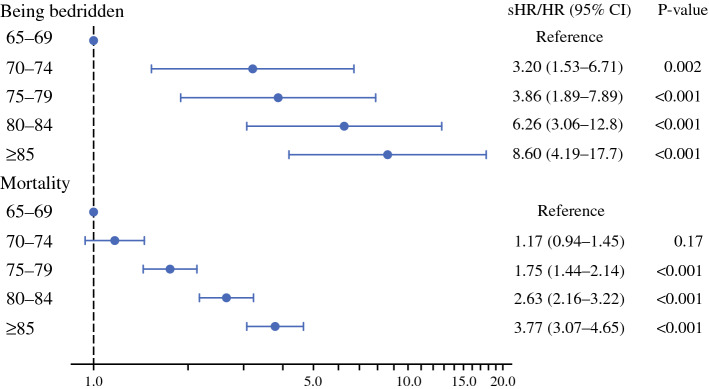
Results of multivariable survival analysis for outcomes following oncologic surgery categorized by age at surgery. *sHR* subdistribution hazard ratio; *HR* hazard ratio; *CI* confidence interval. Hazard ratios for the outcomes were calculated using multivariable survival analysis with adjustment for background factors (patient characteristics and treatment background). For the incidence of bedridden status, we used the Fine–Gray subdistribution hazard model, in which mortality was regarded as a competing risk

The results of multivariable survival analysis are shown in Fig. 2. Patients aged ≥ 70 years had significantly higher incidence of being bedridden than those aged 65–69 years. The subdistribution hazard ratios of the age groups of 70–74, 75–79, 80–84, and ≥ 85 years were 3.20 (95% CI 1.53–6.71), 3.86 (95% CI 1.89–7.89), 6.26 (95% CI 3.06–12.8), and 8.60 (95% CI 4.19–17.7), respectively. Patients aged ≥ 75 years had significantly higher mortality than those aged 65–69 years; the hazard ratios of age groups of 75–79, 80–84, and ≥ 85 years were 1.75 (1.44–2.14), 2.63 (2.16–3.22), and 3.77 (3.07–4.65), respectively. The hazard ratios for background factors are shown in Supplementary Table 2. Surgery site and preoperative care-need level were associated with both outcomes.

**Fig. 3:**
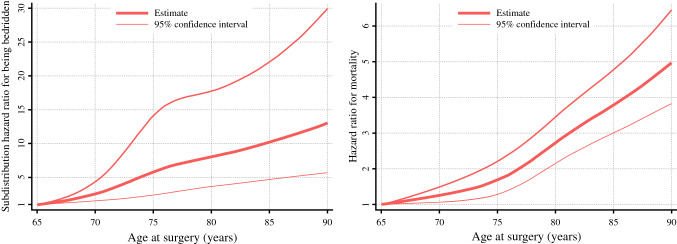
Results of multivariable survival analysis for outcomes following oncologic surgery using a restricted cubic spline function for age at surgery. Hazard ratios for the outcomes were calculated using multivariable survival analysis with adjustment for background factors and a restricted cubic spline with four knots (70, 75, 80, and 85 years). For the incidence of bedridden status, we used the Fine–Gray subdistribution hazard model, in which mortality was regarded as a competing risk. The Y-axis represents the hazard ratio for the outcomes, comparing individuals with an age of 65 years to those at an older age. The estimates are presented with 95% confidence intervals

Figure 3 shows the results of the multivariable survival analysis with restricted cubic spline functions. As the patient aged, the hazard ratios for being bedridden and mortality were higher. Because 95% CIs were consistently above 1 in both analyses, patients aged > 65 years had a significantly higher risk of being bedridden and mortality than those aged 65 years. The curb of the estimated points for being bedridden demonstrated a rapid increase between the ages of 65 and 74 years, and the curb for mortality demonstrated a rapid increase at the age of ≥ 75 years. The associations between age and the outcomes were similar in sensitivity analyses (Supplementary Figs. 2–4).

## Discussion

In the current retrospective study, using Japanese administrative data, we investigated the long-term risk of being bedridden and mortality following major oncologic surgery in approximately 12,000 elderly patients. Older age at surgery was associated with a higher cumulative probability of bedridden status and mortality. Multivariable survival analysis demonstrated an increased risk for being bedridden among patients aged ≥ 65 years, whereas the risk for mortality increased in patients aged ≥ 75 years. To the best of our knowledge, this study is the first to analyze the association between age at surgery and long-term functional outcomes in elderly patients undergoing oncologic surgery.

The dominance of male sex in all age categories corresponds with the worldwide trend; the incidence of cancer in men was reportedly 1.5 times higher than that in women.^31^ Because life expectancy was generally shorter in men than in women^15^ (e.g., United States, 74.2 vs. 79.9 years; Japan, 81.6 vs. 87.7 years in 2020), the number of male patients with malignancy is relatively lesser in the super-elderly population; therefore, the proportion of male sex in patients aged ≥ 85 years was nearly 50%. Older patients had a lower Charlson comorbidity index, because comorbidities can be a major concern when considering indications for oncologic surgery in elderly patients.^2–5^ Older patients with several comorbidities would have been unlikely to undergo surgery. Additionally, the surgical site included in the current study was in line with common cancers globally and in Japan.^2,31,32^ Therefore, we consider that the current results would have generalizability, even if the data were obtained from an administrative database of a district in Japan. Moreover, because the population is aging worldwide and postoperative disability and mortality can likely occur in elderly patients regardless of the region, population, and healthcare system, the current findings would be universally applicable.

In patients aged ≥ 80 years, approximately half of the patients died within the 4-year observation period (Fig. 1), although only patients with few comorbidities and good general condition may have undergone surgery. This is plausible, because the mean expected life years at the age of 85 years was estimated to be approximately 5 years in Japan.^2^ Moreover, approximately a quarter of the patients aged 75–79 years died within the observation period. The current multivariable analysis also demonstrated that the long-term prognosis progressively worsened after age 75 years. Although surgery is supposed to improve long-term prognosis, it should be recognized preoperatively that patients aged ≥ 75 years do not necessarily have good long-term prognosis.

Clinicians provided in-hospital rehabilitation, especially for elderly patients, presumably considering the impaired activities of daily living after surgery.^7,8^ Although rehabilitation could have aided short-term early mobilization, the current analysis with adjustment for preoperative comorbidities and activities of daily living demonstrated that age older than 65 years was substantially associated with poor long-term functional prognosis. Notably, the hazard ratio increased rapidly compared with that for mortality. Patients who were bedridden postoperatively were likely to die within several months (Supplementary Fig. 1); therefore, surgeons should pay close attention to the decline in activities of daily living during outpatient follow-up. Specifically, the cumulative probability of being bedridden reached approximately 25% in patients aged ≥ 80 years at 4 years after surgery. Because the postoperative follow-up for oncologic surgery would generally last for at least 5 years, a surgeon should provide instructions for appropriate rehabilitation during the follow-up, especially for these patients. Additionally, care intervention at home could improve postoperative functional and survival prognoses in elderly patients.^33^

Frailty can be a major concern when considering indications for surgery; therefore, elderly patients who underwent oncologic surgery would be likely to have a better preoperative functional status than those who did not.^2,4^ If all elderly patients underwent oncologic surgery, irrespective of their functional status, the long-term functional and survival outcomes would have been considerably worse than those reported in the current study. Although we presume that clinicians carefully selected candidates for surgery according to preoperative functional status, patient age at surgery was significantly associated with the outcomes. Moreover, the results of sensitivity analyses were similar to those of the main analysis. Irrespective of the surgery site and preoperative care-need level (i.e., background factors significantly associated with both outcomes), age would be a crucially important predictor for the outcomes. We therefore suggest that the current research can provide preoperative explanation for all elderly candidates for oncologic surgery.

Although several procedures can have different effects on long-term outcomes, we had to analyze the procedures together in the current analysis because of the limited number of procedures and their outcomes. Breast and prostate cancers generally have better prognoses than other cancers; these cancers were significantly associated with low mortality in the current analysis (Supplementary Table 2). However, age at surgery was significantly associated with poor functional outcomes even in the current analysis, including these cancers. Furthermore, although partial mastectomy can be achieved in ambulatory settings, breast cancer surgery (including lumpectomy for elderly patients) is generally performed with several-day hospitalization in Japan.^34^ Hence, the inclusion of breast surgery in a Japanese dataset will rarely skew the results. Because analyses stratified by procedures demonstrated similar results to those of the main analysis, the current results can be generalizable irrespective of procedures.

The strength of the current study is the description of long-term functional outcomes. Although the outcome is gaining attention as an important endpoint of cancer treatment, few studies have addressed the functional prognosis.^13^ A Fine–Gray model to account for the competing risk of mortality (i.e., informative censoring) enabled us to conduct the current retrospective longitudinal study. Because surgery does not differ with patient age from a technical point of view,^35^ clinicians would decide the surgical indications in the elderly based mainly on the patient’s preference and general condition. The current study provides important information in the decision-making process, where many elderly patients emphasize functional prognosis.^14^ To handle the age as a continuous variable would be another key of the current study, because age can have nonlinear effects on postoperative outcomes.^36^ The restricted cubic-spline functions clarified that the risk for being bedridden increased considerably in patients aged ≥ 65 years, whereas the risk for mortality increased in patients aged ≥ 75 years. Furthermore, some previous studies have suggested that a specific age may be a barrier for surgical indication.^37^ However, because the risk gradually increased with age (Fig. 3), surgical indication defined at a specific age would be unacceptable.

This study has several limitations. First, the current study did not provide direct evidence of an indication for oncological surgery in elderly patients. We excluded elderly patients who did not undergo oncologic surgery, because we originally planned to investigate long-term functional and survival prognosis following major oncologic surgery in elderly patients and not to compare patients who underwent surgery and those who did not among elderly surgical candidates. Moreover, the comparison according to whether surgery was performed in a retrospective study would include serious confounding factors. However, because surgical indication for elderly patients could not be determined based only on objective preoperative information, such as age, but also on the subjective viewpoints of patients,^14^ the current study can provide useful information on the preoperative decision-making process. Second, we were unable to obtain data on cancer stage, which could be an unmeasured confounding factor. Preoperative treatment and surgical procedures (conventional or scopic surgery) can be used as representatives for the stage. Furthermore, although older patients may have undergone surgery for more advanced cancer, such an association would be part of the effect of age. Thus, we consider that nonadjustment for cancer stage does not diminish the importance of the current study. Finally, the database did not contain information on whether surgery was performed for therapeutic or palliative intent; however, the intent for surgery would be indistinguishable even clinically. Although information on the emergence of surgery also was unavailable, we used the hospital day of surgery (i.e., surgery on the day of admission) as a proxy for the emergence.

## Conclusions

The current, large-scale study revealed that the cumulative probability of being bedridden and mortality increased with a higher age of the patient at surgery. In the multivariable survival analysis with adjustment for background factors, the risk for being bedridden increased considerably in patients aged ≥ 65 years, whereas the risk for mortality increased in patients aged ≥ 75 years. The incidence of malignant tumors is increasing, especially in elderly patients who often emphasize functional prognosis. Therefore, the current study scientifically provides useful information on the preoperative, decision-making process and highlights the importance of long-term observation and maintenance of activities following oncologic surgery among elderly patients.

## Supplementary Information

Below is the link to the electronic supplementary material.

Supplementary file1 (DOCX 639 kb)
