# Supplementary material for: Long-Term Risk of Being Bedridden in Elderly Patients Who Underwent Oncologic Surgery: A Retrospective Study Using a Japanese Claims Database
Source: Ann Surg Oncol. 2023 May 6;30(8):4604–12. doi: 10.1245/s10434-023-13566-5 (PMC10319666; doi:10.1245/s10434-023-13566-5)
Supplement: Supplementary file 1 — Supplementary file1 (DOCX 639 kb) [file 10434_2023_13566_MOESM1_ESM.docx]

**Long-term risk of being bedridden in elderly patients who underwent oncologic surgery: A retrospective study using a Japanese claims database**

Takaaki Konishi, Yusuke Sasabuchi, Hiroki Matsui, Masahiko Tanabe, Yasuyuki Seto, Hideo Yasunaga

**Supplementary Table 1.** Mortality in 657 elderly patients who became bedridden after major surgery categorized by age category at the surgery

**Supplementary Table 2.** Hazard ratios for background factors in multivariable survival analysis for outcomes following oncologic surgery

**Supplementary Figure 1.** Kaplan–Meier curves for mortality in patients after becoming bedridden following oncologic surgery categorized by age at surgery

**Supplementary Figure 2.** Results of multivariable survival analysis for outcomes following oncologic digestive surgery using a restricted cubic spline function for age at surgery

**Supplementary Figure 3.** Results of multivariable survival analysis for outcomes following oncologic non-digestive surgery using a restricted cubic spline function for age at surgery

**Supplementary Figure 4.** Results of multivariable survival analysis for outcomes following oncologic surgery for patients with no preoperative care-needs level using a restricted cubic spline function for age at surgery

**Supplementary Table 1. Mortality in 657 elderly patients who became bedridden after major surgery categorized by age category at the surgery**

|  | Age category at surgery (years) | | | | | | | | | | Total | |  |
| --- | --- | --- | --- | --- | --- | --- | --- | --- | --- | --- | --- | --- | --- |
|  | 65–69 | | 70–74 | | 75–79 | | 80–84 | | ≥85 | |  |  |  |
|  | n=45 | | n=102 | | n=139 | | n=174 | | n=197 | | n=657 | | P-value |
| Mortality | 36 | (80) | 68 | (67) | 92 | (67) | 114 | (66) | 126 | (64) | 436 | (66) | <0.001 |

These outcomes occurred within a median period of 62 (interquartile range, 11–184) days after the patients became bedridden. The Kaplan–Meier curves for the cumulative probabilities of mortality are shown in Supplementary Figure 4.

**Supplementary Table 2. Hazard ratios for background factors in multivariable survival analysis for outcomes following oncologic surgery**

|  | Bedridden | | |  | Mortality | | |
| --- | --- | --- | --- | --- | --- | --- | --- |
|  | sHR* | 95% CI | P-value |  | HR | 95% CI | P-value |
| *Patient characteristics* |  |  |  |  |  |  |  |
| Male sex | 0.91 | 0.66–1.27 | 0.59 |  | 1.15 | 1.02–1.28 | 0.018 |
| Charlson comorbidity index |  |  |  |  |  |  |  |
| 2 | Reference | |  |  | Reference | |  |
| 3 | 0.46 | 0.24–0.87 | 0.018 |  | 1.09 | 0.85–1.39 | 0.50 |
| 4 | 0.56 | 0.30–1.04 | 0.068 |  | 1.09 | 0.85–1.40 | 0.49 |
| 5 | 0.79 | 0.41–1.53 | 0.48 |  | 1.26 | 0.95–1.66 | 0.10 |
| 6 | 0.65 | 0.29–1.44 | 0.29 |  | 1.29 | 0.95–1.75 | 0.11 |
| 7 | 0.70 | 0.20–2.42 | 0.57 |  | 1.81 | 1.24–2.66 | 0.002 |
| 8 | 0.84 | 0.47–1.50 | 0.57 |  | 1.01 | 0.78–1.31 | 0.95 |
| 9 | 0.65 | 0.38–1.12 | 0.12 |  | 1.27 | 1.01–1.61 | 0.045 |
| 10 | 0.57 | 0.33–1.00 | 0.048 |  | 1.19 | 0.94–1.50 | 0.15 |
| 11 | 0.93 | 0.54–1.59 | 0.78 |  | 1.23 | 0.96–1.59 | 0.11 |
| 12 | 0.79 | 0.40–1.55 | 0.49 |  | 1.26 | 0.95–1.67 | 0.12 |
| 13 | 0.73 | 0.33–1.63 | 0.44 |  | 1.63 | 1.20–2.21 | 0.002 |
| 14 | 1.34 | 0.58–3.12 | 0.50 |  | 1.12 | 0.74–1.68 | 0.60 |
| ≥15 | 0.71 | 0.28–1.82 | 0.48 |  | 1.87 | 1.31–2.67 | 0.001 |
| Care-needs level |  |  |  |  |  |  |  |
| None | Reference | |  |  | Reference | |  |
| Support level 1 | 1.48 | 0.75–2.94 | 0.26 |  | 1.92 | 1.50–2.45 | <0.001 |
| Support level 2 | 2.12 | 1.15–3.91 | 0.017 |  | 2.14 | 1.69–2.70 | <0.001 |
| Care-needs level 1 | 2.80 | 1.68–4.66 | <0.001 |  | 2.75 | 2.26–3.35 | <0.001 |
| Care-needs level 2 | 2.48 | 1.34–4.58 | 0.004 |  | 3.79 | 3.08–4.67 | <0.001 |
| Care-needs level 3 | 6.79 | 4.06–11.4 | <0.001 |  | 5.14 | 4.07–6.49 | <0.001 |
| *Treatment background* |  |  |  |  |  |  |  |
| Site of surgery |  |  |  |  |  |  |  |
| Bladder | 3.61 | 1.39–9.35 | 0.008 |  | 0.71 | 0.46–1.10 | 0.13 |
| Breast | 4.83 | 1.78–13.1 | 0.002 |  | 0.21 | 0.12–0.35 | <0.001 |
| Colorectum | 4.08 | 1.67–9.97 | 0.002 |  | 0.91 | 0.60–1.38 | 0.65 |
| Stomach | 3.12 | 1.30–7.46 | 0.011 |  | 1.23 | 0.81–1.87 | 0.32 |
| Liver | 3.26 | 0.69–15.4 | 0.14 |  | 0.95 | 0.55–1.62 | 0.84 |
| Lung | 3.63 | 1.25–10.6 | 0.018 |  | 1.53 | 0.97–2.42 | 0.070 |
| Pancreas | 4.58 | 1.78–11.7 | 0.002 |  | 2.35 | 1.51–3.68 | <0.001 |
| Prostate | 1.90 | 0.49–7.39 | 0.36 |  | 0.04 | 0.01–0.13 | <0.001 |
| Uterus | 3.99 | 0.87–18.2 | 0.074 |  | 0.57 | 0.29–1.14 | 0.11 |
| Preoperative treatments |  |  |  |  |  |  |  |
| Chemotherapy | 1.16 | 0.62–2.16 | 0.65 |  | 1.25 | 1.01–1.54 | 0.037 |
| Radiotherapy | 1.87 | 0.67–5.22 | 0.23 |  | 2.49 | 1.76–3.51 | <0.001 |
| Surgery on the day of admission | 1.11 | 0.56–2.18 | 0.77 |  | 1.29 | 0.98–1.70 | 0.067 |
| Scopic surgery | 0.88 | 0.59–1.32 | 0.54 |  | 0.44 | 0.38–0.52 | <0.001 |
| In-hospital rehabilitation | 1.07 | 0.80–1.44 | 0.65 |  | 1.35 | 1.21–1.51 | <0.001 |

Abbreviations: sHR, sub-distribution hazard ratio; HR, hazard ratio; CI, confidence interval

*For the incidence of bedridden status, we used the Fine–Gray sub-distribution hazard model, in which mortality was regarded as a competing risk.

Bladder, breast, colorectum, gastric, lung, and pancreatic cancer was associated with poor functional outcomes. Pancreatic cancer showed significantly higher mortality, whereas breast and prostate cancer showed lower mortality compared to other sites. Preoperative care-needs level was associated with both poor functional and survival prognosis. Male sex showed poor survival prognosis presumably because men generally have a shorter life expectancy than women. Preoperative chemotherapy, preoperative radiotherapy, and conventional surgery were also associated with poor survival prognosis presumably because they reflect an advanced stage of cancer.

**Supplementary Figure 1. Kaplan–Meier curves for mortality in patients after becoming bedridden following oncologic surgery categorized by age at surgery**


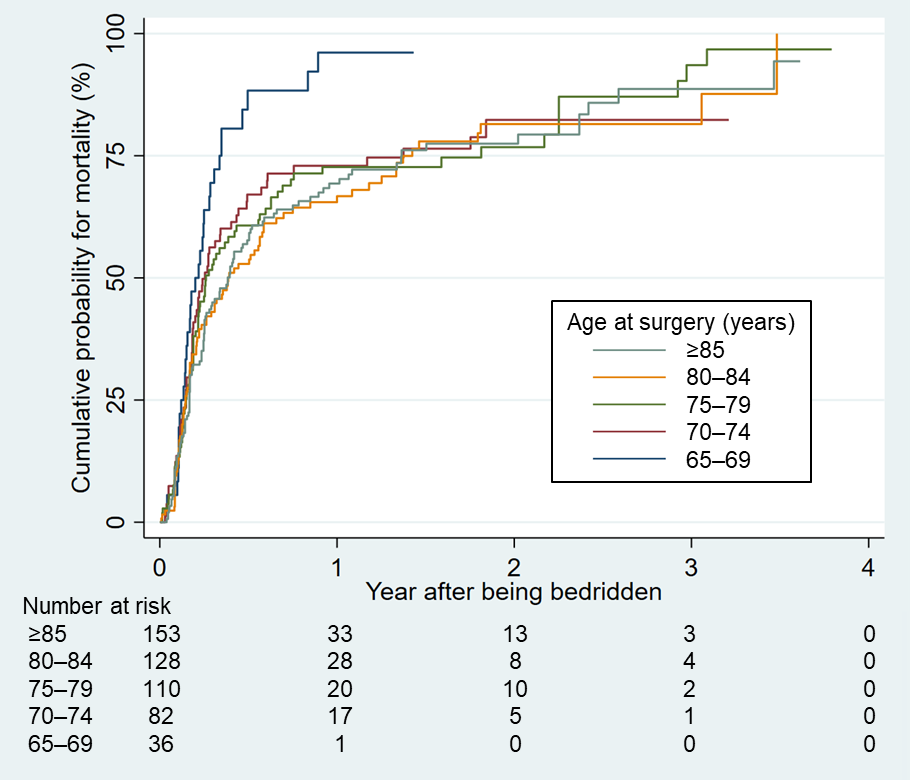


Patients aged 65–69 years at the surgery died earlier than those aged ≥70 years presumably because patients who became bedridden despite their young age inherently had poor general conditions.

**Supplementary Figure 2. Results of multivariable survival analysis for outcomes following oncologic digestive surgery using a restricted cubic spline function for age at surgery**


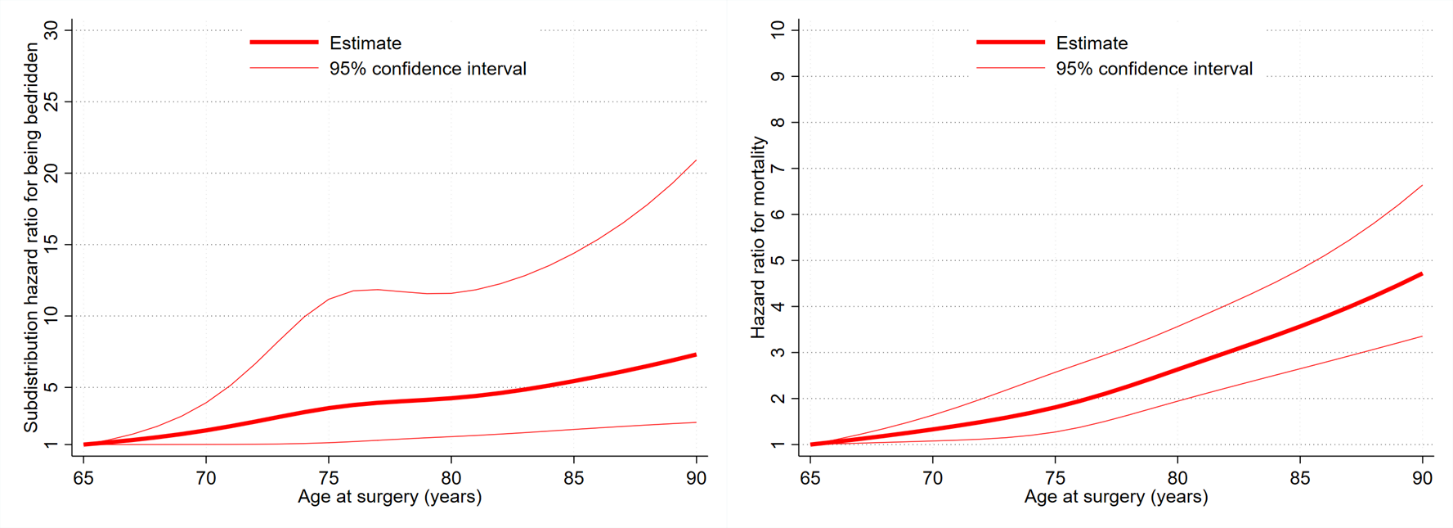


Hazard ratios for the outcomes were calculated using multivariable survival analysis with adjustment for background factors and a restricted cubic spline with four knots (70, 75, 80, and 85 years). For the incidence of bedridden status, we used the Fine–Gray sub-distribution hazard model, in which mortality was regarded as a competing risk. The Y-axis represents the hazard ratio for the outcomes, comparing individuals with an age of 65 years to those at an older age. The estimates are presented with 95% confidence intervals.

Digestive surgery was defined as surgery for the colorectum, stomach, liver, and pancreas.

**Supplementary Figure 3. Results of multivariable survival analysis for outcomes following oncologic non-digestive surgery using a restricted cubic spline function for age at surgery**


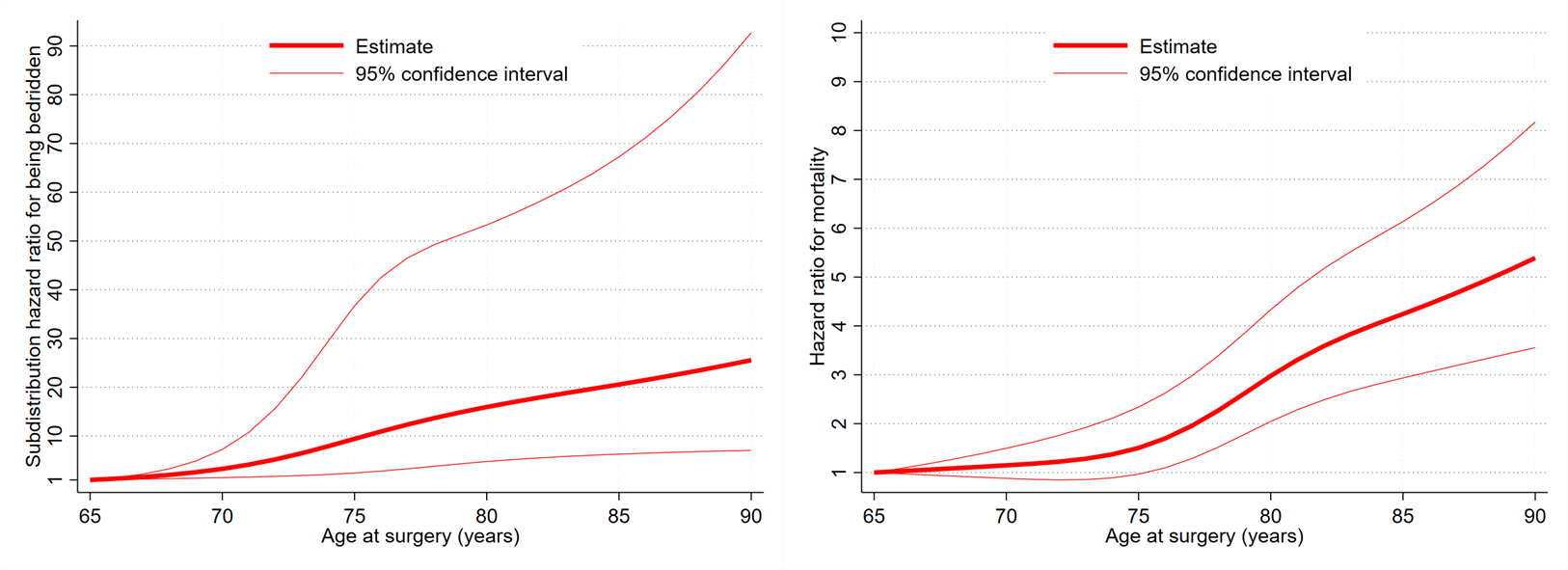


Hazard ratios for the outcomes were calculated using multivariable survival analysis with adjustment for background factors and a restricted cubic spline with four knots (70, 75, 80, and 85 years). For the incidence of bedridden status, we used the Fine–Gray sub-distribution hazard model, in which mortality was regarded as a competing risk. The Y-axis represents the hazard ratio for the outcomes, comparing individuals with an age of 65 years to those at an older age. The estimates are presented with 95% confidence intervals.

Non-digestive surgery was defined as surgery for the bladder, breast, lung, prostate, and uterus.

**Supplementary Figure 4. Results of multivariable survival analysis for outcomes following oncologic surgery for patients with no preoperative care-needs level using a restricted cubic spline function for age at surgery**


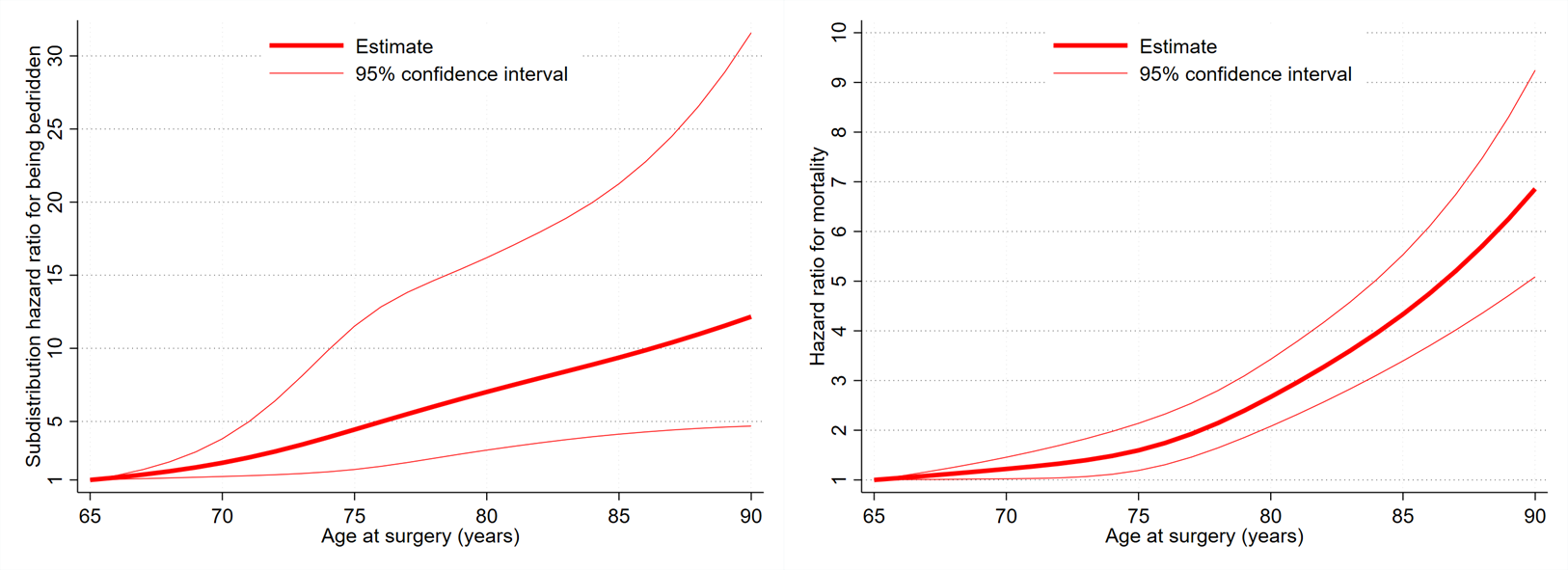


Hazard ratios for the outcomes were calculated using multivariable survival analysis with adjustment for background factors and a restricted cubic spline with four knots (70, 75, 80, and 85 years). For the incidence of bedridden status, we used the Fine–Gray sub-distribution hazard model, in which mortality was regarded as a competing risk. The Y-axis represents the hazard ratio for the outcomes, comparing individuals with an age of 65 years to those at an older age. The estimates are presented with 95% confidence intervals.
